# Supplementary material for: Structural insight into catalytic mechanism of PET hydrolase
Source: Nat Commun. 2017 Dec 13;8:2106. doi: 10.1038/s41467-017-02255-z (PMC5727383; doi:10.1038/s41467-017-02255-z)
Supplement: Supplementary file 1 — Supplementary Information [file 41467_2017_2255_MOESM1_ESM.doc]

Supporting information

**Structural insight into catalytic mechanism of PET hydrolase**

Xu Han^1^*, Weidong Liu^1^*, Jian-Wen Huang^2^*, Jiantao Ma^1,3^, Yingying Zheng^1^, Tzu-Ping Ko^4^, Limin Xu^2^, Ya-Shan Cheng^2^, Chun-Chi Chen^1^, and Rey-Ting Guo^1^

^1^Industrial Enzymes National Engineering Laboratory, Tianjin Institute of Industrial Biotechnology, Chinese Academy of Sciences, Tianjin 300308, China

^2^AsiaPac Biotechnology Co., Ltd, Dongguan 523808, China

^3^School of Biotechnology, Jiangnan University, Wuxi 214122, China

^4^Institute of Biological Chemistry, Academia Sinica, Taipei 11529, Taiwan

*These authors contribute equally.

Corresponding authors: R.-T.G. (Email: guo_rt@tib.cas.cn)

**Supplementary table**

**Supplementary Table 1.** Mutagenesis oligonucleotides

|  | Sequence (5’🡪 3’) |
| --- | --- |
| S131A | ATGGGTGTTATGGGATGGGCTATGGGTGGCGGGGGTTCCT |
| R103G | CAACAAATGGCTGCTTTGGGTCAAGTTGCTTCCTTGAACG |
| C174S | CCTACTTTGATTTTTTCCGCTGAAAACGATTCTATTGCT |
| C210S | AACGGAGGATCACATTCATCCGCTAACTCAGGAAACTCA |
| W156A | GCTGCTCCACAAGCTCCTGCTGATTCCTCCACTAACTTT |
| S185H | TCTATTGCTCCAGTTAACTCTcacGCTTTGCCAATATACGATTCT |
| I179A | GCTTGTGAAAACGATTCTgctGCTCCAGTTAACTCTTCCGCTTT |
| W130A | AGAATGGGTGTTATGGGAGCTTCTATGGGTGGCGGGGGTT |
| W130H | AGAATGGGTGTTATGGGACACTCTATGGGTGGCGGGGGTT |
| M132A | ATGGGTGTTATGGGATGGTCTgctGGTGGCGGGGGTTCCTTGATT |
| Y58A | ATTGCTATTGTTCCAGGTgctACTGCTAGACAATCCTCAATTAAG |
| T59A | GCTATTGTTCCAGGTTACgctGCTAGACAATCCTCAATTAAGTGG |

**Supplementary Table 2.** Data collection and refinement statistics of PETase crystals*

|  | **WT** | **S131A** | **DM** | **DM-HEMT** | **DM-pNP** |
| --- | --- | --- | --- | --- | --- |
| **Data collection** |  |  |  |  |  |
| Space group | P2_1_2_1_2_1_ | C2 | P2_1_2_1_2_1_ | P2_1_2_1_2_1_ | P2_1_2_1_2_1_ |
| Cell dimensions |  |  |  |  |  |
| *a*, *b*, *c* (Å) | 63.48, 69.22, 105.04 | 114.19, 50.33, 40.98 | 50.95, 51.62, 84.59 | 50.90, 51.28, 84.11 | 50.78, 51.53, 84.37 |
| β (°) |  | 92.24 |  |  |  |
| Resolution (Å) | 25-1.58  (1.64-1.58)** | 25-1.40  (1.45-1.40) | 25-1.55  (1.61-1.55) | 25-1.30  (1.35-1.30) | 25-1.20  (1.24-1.20) |
| *R*_merge_ (%) | 3.9 (7.6) | 3.6 (20.7) | 7.6 (49.1) | 5.0 (34.3) | 5.0 (21.2) |
| *I* / σ*I* | 42.2 (28.8) | 57.4 (10.1) | 18.9 (2.7) | 29.7 (3.1) | 32.6 (7.2) |
| Completeness (%) | 99.8 (100) | 99.7 (100) | 99.9 (100) | 99.6 (98.2) | 99.8 (98.9) |
| Redundancy | 5.4 (5.4) | 5.7 (5.8) | 4.7 (4.6) | 6.9 (5.9) | 8.0 (7.6) |
|  |  |  |  |  |  |
| **Refinement** |  |  |  |  |  |
| Resolution (Å) | 25-1.58  (1.62-1.58) | 25-1.40  (1.44-1.40) | 25-1.55  (1.59-1.55) | 25-1.30  (1.33-1.30) | 25-1.20  (1.23-1.20) |
| No. reflections | 88161 (6622) | 43291 (3354) | 31429 (2396) | 51930 (3894) | 66256 (4928) |
| *R*_work_ / *R*_free_ | 13.7 (13.3)/ 16.1 (18.7) | 13.4 (15.6)/  15.8 (17.7) | 13.8 (20.8)/ 16.9 (26.4) | 11.7 (19.3)/ 14.5 (21.9) | 9.8 (10.3)/ 12.4 (14.0) |
| No. atoms |  |  |  |  |  |
| Protein | 5813 | 1931 | 1935 | 1931 | 1945 |
| Ligand/ion |  | 21 | 38 | 32 | 30 |
| Water | 1124 | 190 | 235 | 244 | 365 |
| *B*-factors |  |  |  |  |  |
| Protein | 10.5 | 16.5 | 12.9 | 11.4 | 9.4 |
| Ligand/ion |  | 37.3 | 42.6 | 30.9 | 25.4 |
| Water | 23.0 | 30.1 | 26.1 | 30.1 | 27.3 |
| R.m.s. deviations |  |  |  |  |  |
| Bond lengths (Å) | 0.010 | 0.011 | 0.011 | 0.010 | 0.011 |
| Bond angles (°) | 1.51 | 1.50 | 1.50 | 1.50 | 1.49 |
| Ramachandran plot [%] |  |  |  |  |  |
| Most favored | 98.1 | 98.5 | 98.1 | 98.1 | 98.1 |
| Additionally allowed | 1.9 | 1.5 | 1.9 | 1.9 | 1.9 |

* One crystal used for the structure.

** Values in parentheses are for the highest resolution shell.

**Supplementary figures**


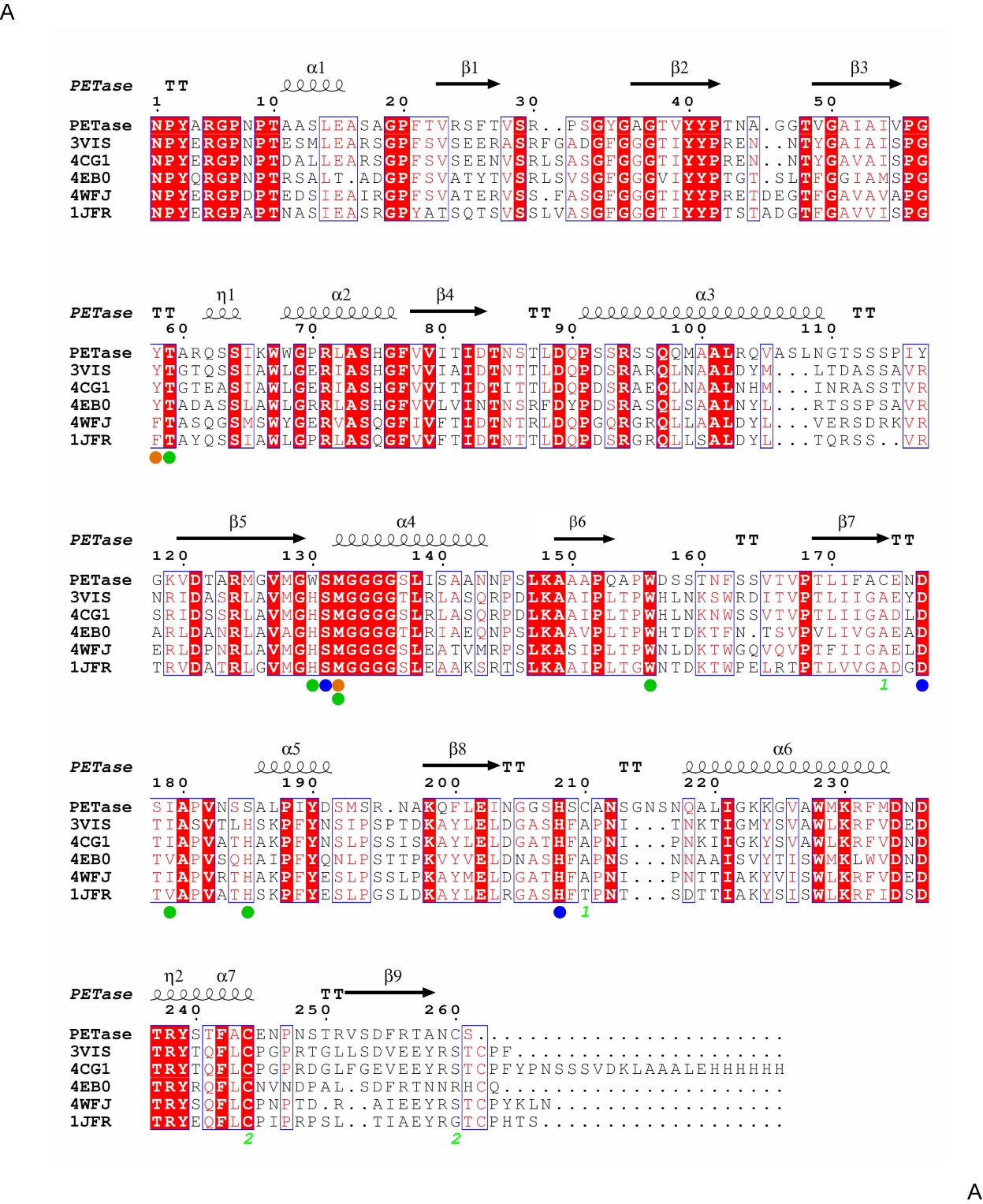


**Supplementary figure 1.** Sequence alignment of *I. sakaiensis* PETase (PETase) together with several other enzymes exhibiting PET-hydrolytic activity. 3VIS, *Thermobifda alba* cutinase; 4CG1, *Thermobifida fusca* cutinase; 4EB0, leaf-branch compost bacteria cutinase; 4WFJ, *Sacharomonospora viridis* cutinase; 1JFR, *Streptomyces exfoliatus* lipase. Residues composing the catalytic triad are labeled by blue dots, and residues involved in oxyanion hole are labeled by orange dots. Residues involving in HEMT, pNP, and modeled PET interactions are indicated by green dots. Disulfide bond-forming cysteine pairs are indicated by green numbers.


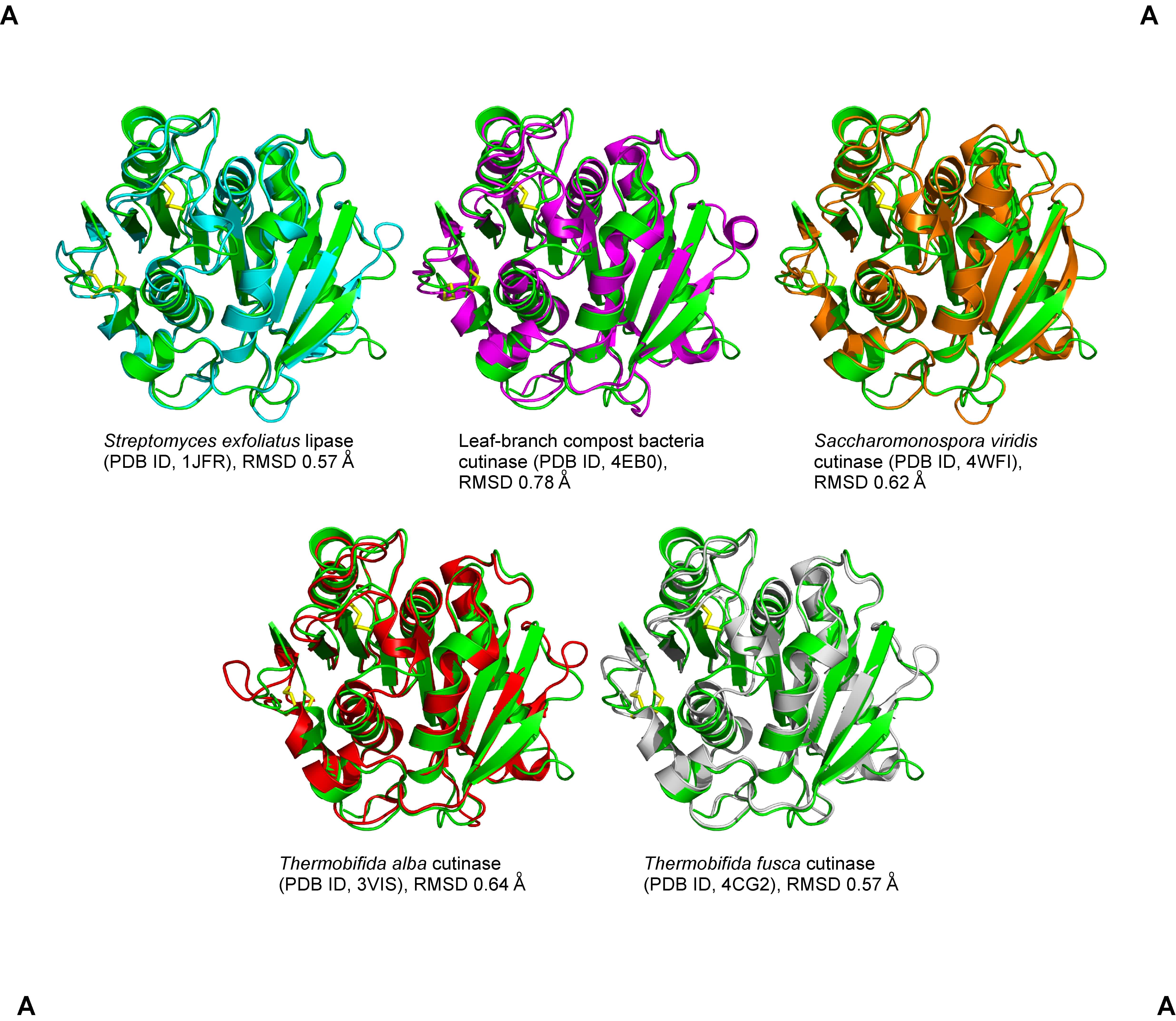


**Supplementary figure 2.** Structural superimpositions of PETase and other enzymes with PET-hydrolytic activity. Disulfide bridges are presented in yellow sticks. PETase structure was shown in green.


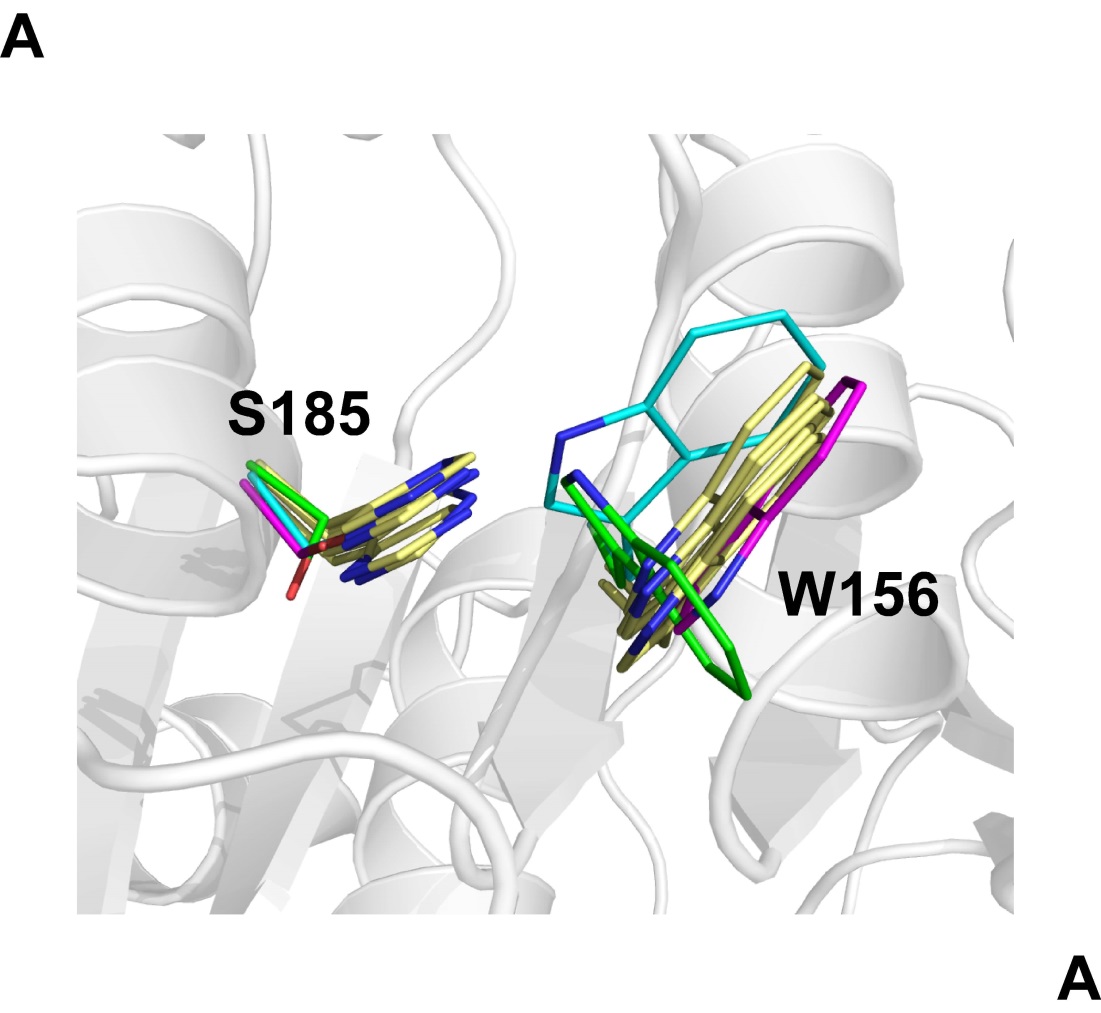


**Supplementary figure 3.** Corresponding residues of W156 and S185 in PETase homologous structures. PETase and other homologous structures (PDB ID shown in Supplementary Fig. 1) are superimposed. W156 and S185 of A, B, and C chain of PETase are colored in green, cyan, and magenta. The corresponding residues in the other structures are in yellow color. The protein structure of PETase (A chain) is shown in a cartoon model.


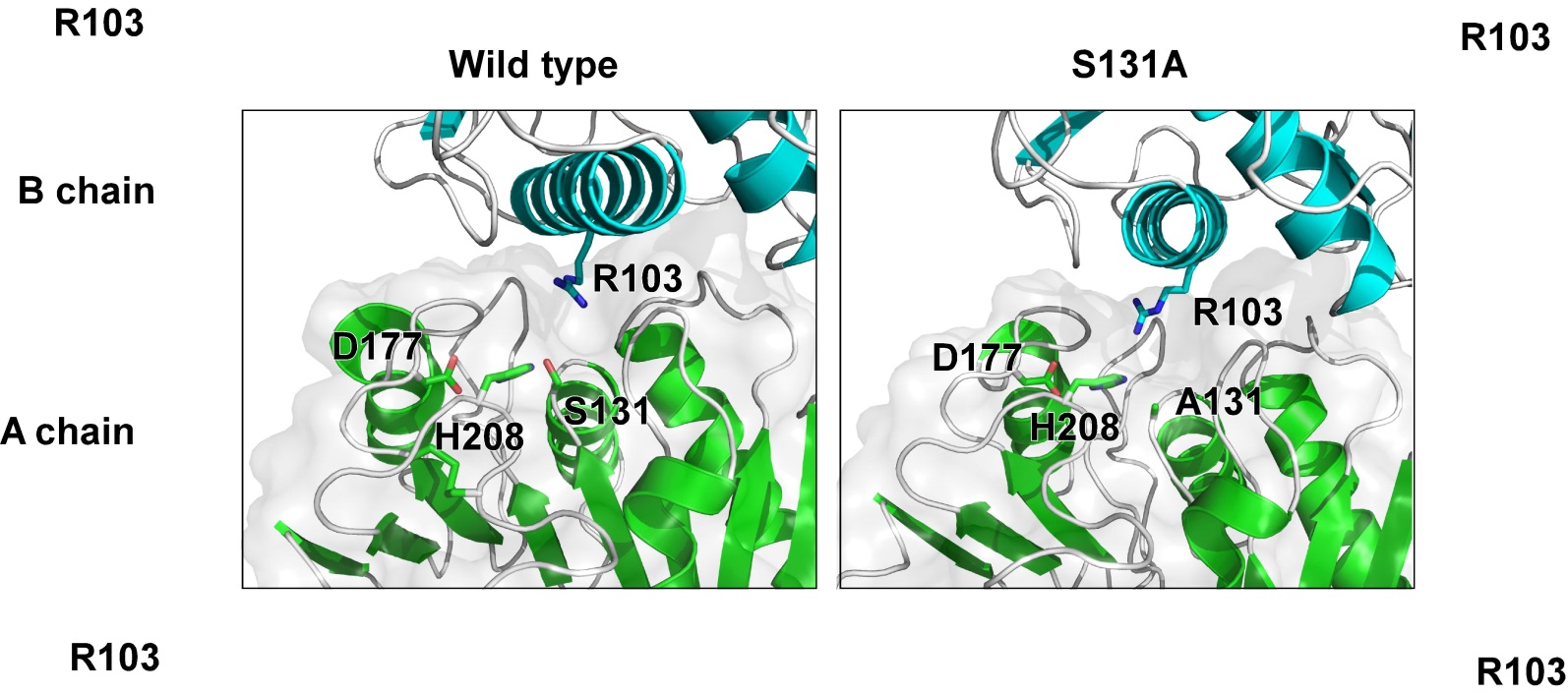


**Supplementary figure 4.** R103 side chain occupies putative active site cleft. Two neighboring polypeptide chains in WT (left) and S131A (right) crystals are shown as cartoons in green and cyan. Protein surface of the green chain is also shown. The side chains of catalytic triad and R103 are shown as sticks. R103 side chain in cyan chain stretches into the shallow cavity above the 131^st^ residue in green chain.


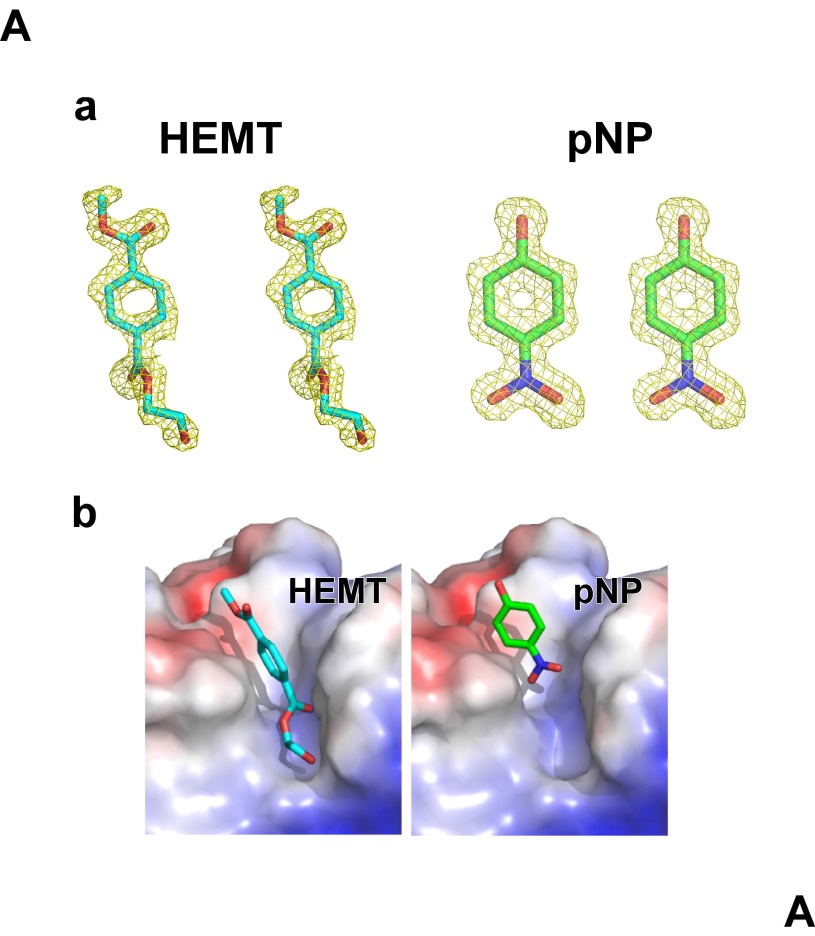


**Supplementary figure 5.** (**a**) *F*_o_-*F*_c_ electron omit maps of bound ligands in PETase complex structures are contoured at 3.0 σ and presented in stereo-view. (**b**) Ligands in DM-HEMT and DM-pNP are shown as sticks. The proteins are covered by electrostatic surfaces.


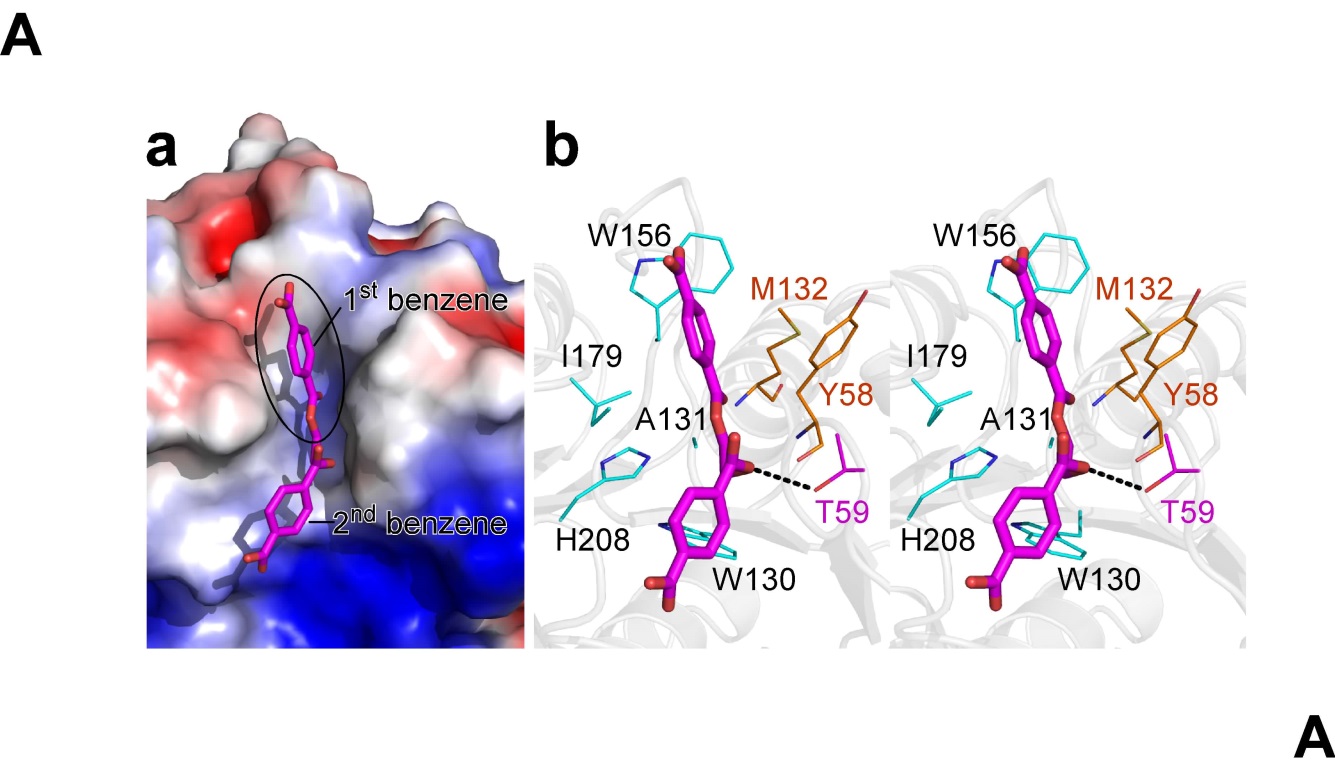


**Supplementary figure 6.** PET docking result. (**a**) Electrostatic surface presentation of DM-HEMT structure docked with a PET molecule (magenta stick). Two phenyl ring of PET are indicated. (**b**) Stereo view of PET-PETase interaction network. The PET molecule is presented as in **a.** Residues participating in PET binding are shown as thin sticks. The oxyanion hole-forming residues are in orange, and T59 that might form H-bond is shown in magenta.


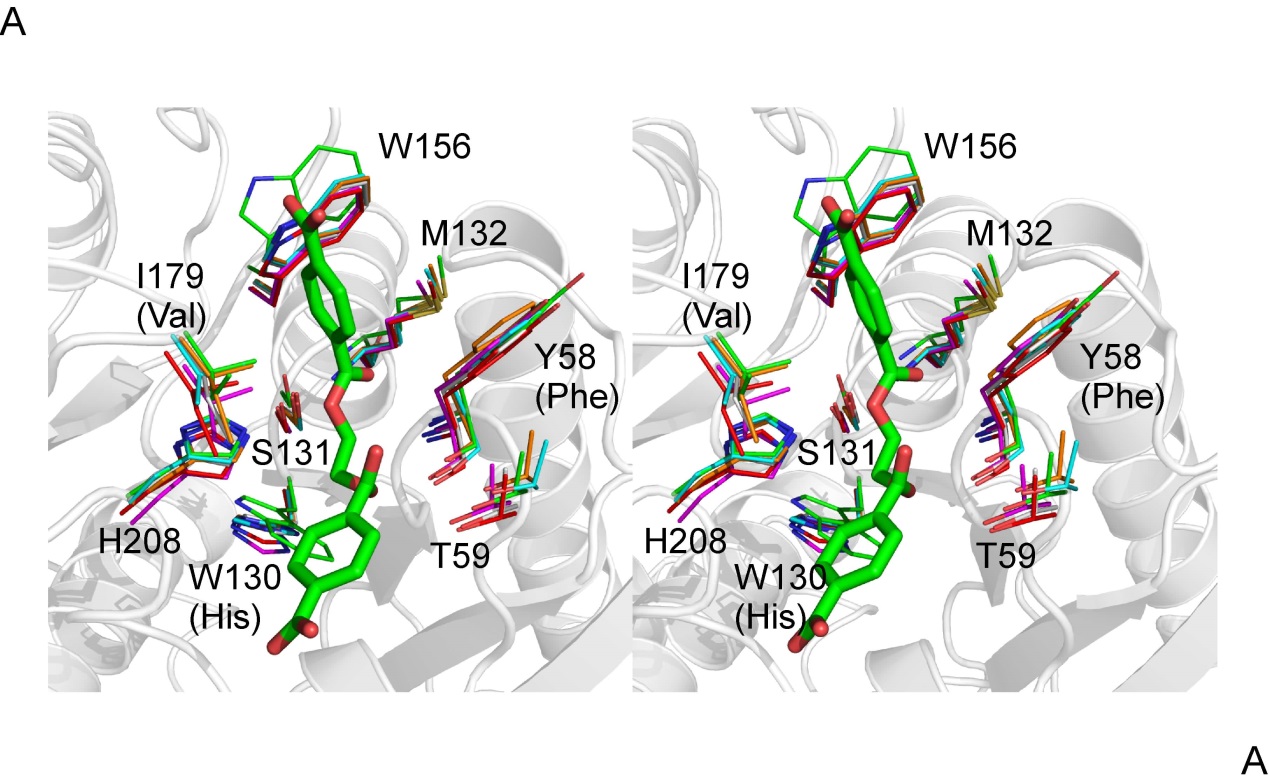


**Supplementary figure 7.** Substrate-interacting network of PETase and homologous enzymes. PETase (Chain A, green) and other homologous structures are superimposed. PET molecule is derived from docking structure (Supplementary Fig. 6). PET-interacting residues in PETase and corresponding residues in other enzymes were shown (color scheme as shown in Supplementary Fig. 2). The shown residues are strictly conserved or semi-conserved (variable residues in other enzymes are noted in parentheses).


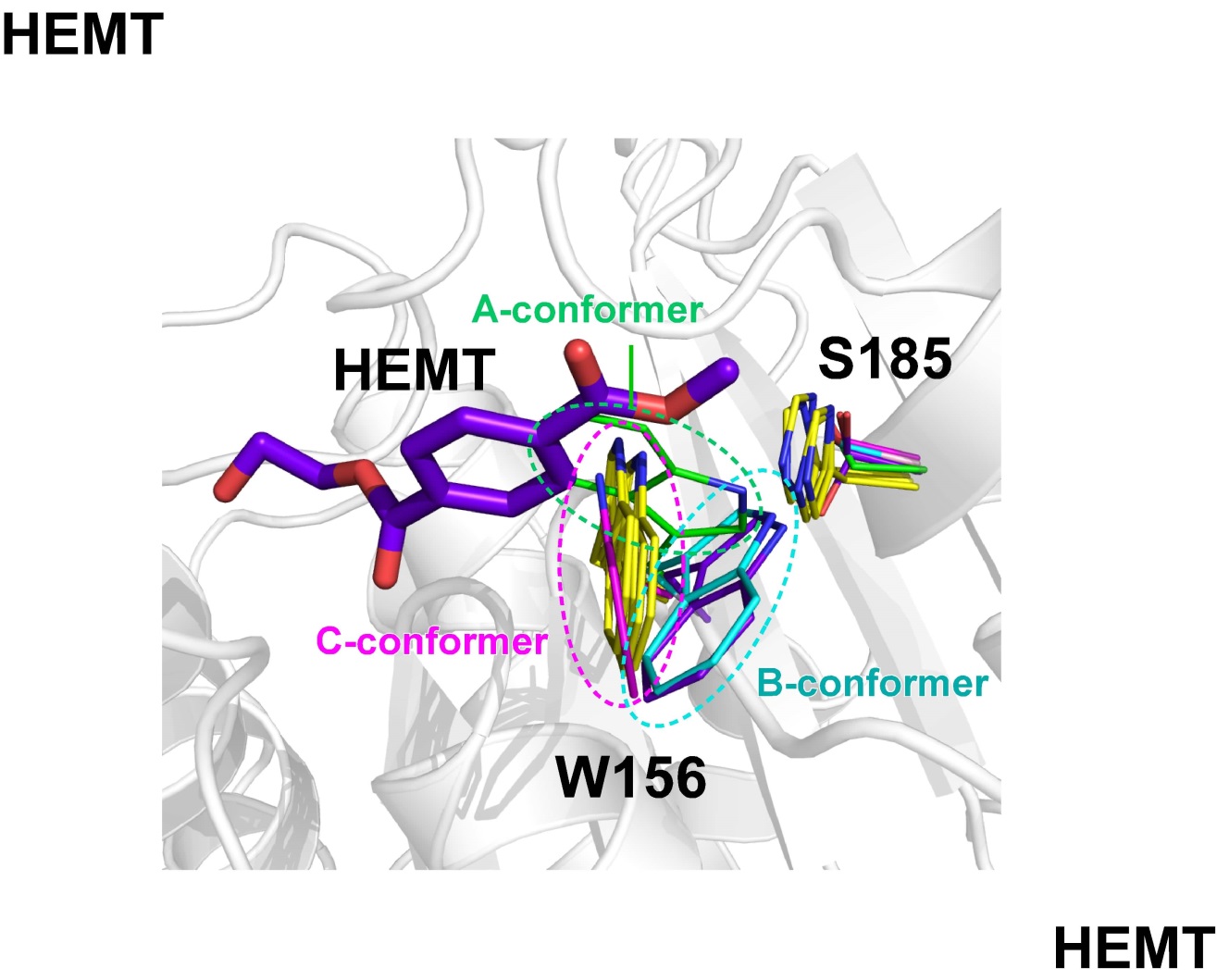


**Supplementary figure 8.** W156 in “B-conformation” is the favorable form of substrate-binding. Superimposition of apo-form PETase, DM-HEMT, and other homologous enzymes. S185 and W156 (in A-, B-, and C- conformation) of PETase are shown. The corresponding residues of S185 and W156 in other homologous enzymes are also shown. Structure presentation and color scheme are the same as in Supplementary figure 3. DM-HEMT is in purpleblue color, and HEMT is in a stick model.
